# Uranium-Platinum System

**DOI:** 10.6028/jres.064A.009

**Published:** 1960-02-01

**Authors:** J. J. Park, D. P. Fickle

## Abstract

The phase diagram of the uranium-platinum system was constructed from data obtained by thermal analysis, metallographic examination, and X-ray diffraction. The system is characterized by four intermetallic compounds: UPt, formed peritectoidally at 961° C; UPt_2_, formed peritectically at 1,370° C; UPt_3_, melting congruently at 1,700° C; and UPt_5_, formed peritectically at 1,460° C. One eutectic occurs at 1,005° C and 12 a/o platinum, and a second at 1,345° C and 87.5 a/o platinum. The maximum solubilities are 4.5 a/o uranium in platinum and 5 a/o platinum in gamma-uranium. Platinum lowers the gamma-beta uranium transformation to 705° C and the beta-alpha transformation to 589° C.

## 1. Introduction

The study of the uranium-platinum system, reported herein, represents the completion of the initial phase of a program designed to establish the binary phase relationships between uranium and the platinide metals; the data from these studies will be correlated in the light of modern alloy theory, as an extension of earlier efforts in this field [1].^1^ The data upon which this proposed uranium-platinum diagram was constructed were obtained primarily from thermal analysis, X-ray diffraction, and metallographic studies.

## 2. Previous Work

A survey of the literature up to 1948 revealed that information on the constitution of the uranium-platinum system was meager [2]. A more recent compilation of the literature by Sailer and Rough [3] in 1955 reported the unpublished information that the UPt_2_ compound had the hexagonal *C*–36 type MgNi_2_ structure. It was also claimed that the beta uranium phase had been retained at room temperature by quenching alloys of low platinum content. Heal and Williams [4] later reported the compound UPt_3_; however, their investigation was limited to preparing this particular compound.

## 3. Preparation and Analysis of Alloys

The component metals consisted of uranium of about 99.9 percent purity (Mallinckrodt biscuit) and 99.5 percent pure platinum sponge.^2^ The platinum sponge was compressed into small pellets with a hydraulic press prior to preparing the charge.

Alloys in the range 0 to 45 a/o platinum were induction melted under vacuum using beryllia crucibles. Because an alloy of 54.8 a/o platinum showed evidence of reaction with the refractory, alloys in the composition range of 45 to 99.5 a/o platinum were prepared by arc melting under an atmosphere of helium. However, additional melts showed that alloys of greater than 75 a/o platinum did not react with beryllia crucibles.

Chemical analyses were made for one constituent on the as melted alloys. When determining the platinum content, alloys containing less than 6 a/o platinum were assayed spectrophotometrically, and alloys containing more than 6 a/o platinum were assayed gravimetrically. The uranium determinations were made spectrophotometrically for alloys with less than 12 a/o uranium and volumetrically for alloys containing more than 12 a/o uranium. The difference between the nominal and analyzed compositions was small, being less than 0.5 w/o when the alloys were prepared by arc melting and less than this when induction melted. The compositions of the alloys used in this investigation are given in table 1.

## 4. Procedures

### 4.1. Thermal Analysis

Thermal arrests were determined from time-temperature curves, the samples being heated in a molybdenum-wound resistance furnace. The accessory apparatus included an electronic controller to maintain uniform heating and cooling rates and an electronic recorder to plot automatically the heating and cooling curves. The thermal curves were obtained from specimens weighing approximately 100 g, heated and cooled under vacuum (about 10*μ*) at a rate of approximately 3° C/min. A nickel cylinder, which was originally used as a thermal reservoir, limited the maximum temperature to about 1,400° C; a molybdenum cylinder was later used as a thermal reservoir. The maximum temperature of the furnace was about 1,550° C. Temperature determinations were made with an annealed Pt versus Pt–10 percent Rh thermocouple, compared prior to use against a reference thermocouple calibrated at NBS. Under experimental conditions, temperature measurements are estimated to be accurate to ±2° C.

The thermal arrests of all alloys were derived from the cooling curves. When determining liquidus temperatures in alloys of greater than 74.6 a/o platinum above 1,500° C, thermal analyses were made in an induction furnace under vacuum, and temperature measurements were made with an annealed molybdenum-tungsten thermocouple contained in a beryllia protection tube. This thermocouple was calibrated against the melting point of the platinum sponge; it is believed to be accurate to within ±10° C under the experimental conditions. For these determinations, a premelted alloy was remelted by induction so that the thermocouple and its protection tube could be inserted from above. A cooling curve was obtained on an electronic recorder while the decrease in temperature was achieved by reducing the input to the converter gradually and uniformly by means of a motor-driven gear and chain drive.

Thermal analyses of alloys in the range 0 to 45 and 75 to 100 a/o platinum were made with the alloys contained in beryllia crucibles. Since alloys in the range 45 to 75 a/o platinum sometimes showed a reaction with the beryllia crucibles, thermal analyses on these alloys were made with the samples resting on either thoria or beryllia plates and heated by induction. Whenever metallographic observation indicated that contamination was excessive, the results were discarded. For some of the high temperature determinations, a filament furnace in which the specimen was heated by its electrical resistance was employed, with temperatures being determined with an optical pyrometer to an accuracy of ± 10° C. Values thus obtained with no possibility of contamination by a crucible were considered to be reliable data. Only one temperature determination, either the melting point or the decomposition temperature, could be made with the optical pyrometer. The observed temperatures were used, no correction being made for the emissivity of the specimens.

### 4.2. Metallographic Analysis

The specimens for metallographic analysis were mounted in Bakelite and then rough ground on a series of silicon carbide papers, the final paper being 000 grit. The specimens were then finished on a wet broadcloth lap with either levigated alumina or with 1-*μ* diam diamond powder as the abrasive. In many instances, the uranium-rich alloys required a final electrolytic polish; the electrolytic solution consisted of 5 parts orthophosphoric acid, 8 parts ethylene glycol, and 8 parts ethyl alcohol. This final polish required 3 to 6 immersions of 15 sec each at 30 v and 10 amp/cm^2^.

The structures of the uranium-rich alloys were developed by electrolytic etching consisting of 2 to 4 immersions of 10 sec each at 5 v and 1 amp/cm^2^ in a 10 percent chromic acid solution. Alloys in the range 50 to 75 a/o platinum exhibited a high degree of resistance to chemical attack. Immersion periods as long as 5 min in an aqueous solution of 60 percent nitric acid were often required to obtain a satisfactory etch. Some platinum-rich alloys were etched by immersion in aqua regia, but, as this reaction was not easily controlled, an electrolytic etching method was developed using alternating current and a solution of 5 percent sodium cyanide and employing 2 to 4 immersions of 20 sec each at 10 v and 10 amp/cm^2^.

All of the alloys were homogenized prior to quenching from various temperatures for subsequent metallographic examination. The samples were sealed, under helium atmosphere, in high-silica glass tubes, homogenized for 8 days at temperature, and furnace cooled. Alloys in the composition range 0 to 55 a/o platinum were homogenized at 800° C, those in the range 55 to 75 a/o at 950° C, and those in the range 75 to 99.5 a/o at 1,000° C. The examination of samples from the initial heat treatments revealed that, in the composition range of 55 to 65 a/o platinum, a large amount of contamination from the silica tubing had occurred. To avoid this, alloys for subsequent heat treatment were wrapped in molybdenum sheet prior to sealing in the silica tubing; no molybdenum contamination was detected by chemical tests. The homogenized specimens were subsequently sealed individually in silica tubing, reheated to selected temperatures, and quenched in ice water, using the nickel-block technique [5]. Quenching temperatures were measured with a Pt versus Pt–10 percent Rh thermocouple, previously calibrated against a standard thermocouple, which under the experimental conditions was accurate to ±2° C. In determining the solubility of platinum in uranium, the specimens were heated into the gamma range, then cooled to the desired temperature, and quenched. When quenching to determine the solidus line, the samples were quenched from the highest temperature attained. To determine the uranium solubility in platinum, homogenized specimens were heated to above 1,250° C before cooling to the quenching temperature. For temperatures above 1,250° C, the specimens were quenched when the desired temperature was attained on heating.

Because several of the phases were observed to be optically active, the phase boundaries were located by using polarized light. The alloys were examined under crossed Nicols and the characteristic extinctions of each of the various phases observed. In some cases, no extinctions were observed when the stage of the microscope was rotated through 360°. In others, when the stage was rotated through 360°, two extinctions were observed and the phase was designated as two-fold active. In still other instances four extinctions were apparent as the specimen was rotated through 360°, and this phase was designated as four-fold active.

### 4.3. X-Ray Analysis

The specimens which were used for the metallographic studies were subsequently placed in an X-ray diffractometer, and their room temperature diffraction patterns obtained by the use of either Cu-K*α* or Co-K*α* radiation. Powder patterns and film techniques were subsequently employed to supplement the diffractometer data. By the application of these techniques, and the utilization of the disappearing- phase method of analysis, it was possible to approximate the composition at which phase boundaries occurred. These results were then correlated with the thermal and metallographic data.

### 4.4. Hardness Determinations

The hardnesses of the various microconstituents were determined by the static indentation method, using a Bergsman hardness tester. The applied load ranged from 1 to 10 g, with an indentation time of 10 sec, and measurements were made at × 750. The results were then converted to diamond pyramid hardness (DPH), kg/mm^2^.

## 5. Results

### 5.1. Thermal Analysis Data

The results of the thermal analyses are given in table 2, with the “arrest points,” as determined from cooling curves, listed. These data indicate that additions of platinum to uranium lower the melting points of uranium alloys to a projected minimum of 1,005° C at about 12 a/o platinum. From this point, the melting points of the uranium-platinum alloys rise to a maximum of 1,700° C at 75 a/o platinum. From this maximum the liquidus temperatures drop rapidly to an indicated low of 1,345° C at about 87.5 a/o platinum and then rise rapidly to the melting point of the platinum sponge at 1,760° C.

The transformation temperatures of the allotropic forms of uranium were affected by the addition of platinum. The gamma-beta transformation temperature of uranium was lowered from 762° C to 705° C by the addition of platinum. This arrest temperature was fairly constant for alloys containing from 5 to 33 a/o platinum. The beta-alpha transformation temperature was similarly lowered from 657° C to 589° C in the composition range of 0.5 to 13 a/o platinum. A suppressing effect was noted in alloys containing from 13 to 33 a/o platinum, resulting in the beta-alpha transformation sometimes occurring as low as 567° C. Greater variation in the beta-alpha uranium transformation temperature than in the gamma-beta uranium transformation temperature is evident from the thermal analysis data. These data may indicate the greater likelihood of retaining the beta- rather than the gamma-uranium phase on quenching.

In the composition range of 0 to 75 a/o platinum, liquidus determinations show that the melting points of the alloys decrease from 1,131° C to about 1,005° C over a composition range of 0 to 12 a/o platinum. The arrest at about 1,005° C occurred in alloys containing up to 33.6 a/o platinum, thus being indicative of a eutectic reaction. A second reaction horizontal was similarly observed to occur near 961° C in alloys containing up to 44.7 a/o platinum, thus indicating the solid state decomposition of an intermetallic compound. A third horizontal was observed at about 1,370° C in alloys of 60.4 and 68.2 a/o platinum, and this was considered to be indicative of the formation of a second intermetallic compound.

The melting point of the 75 a/o platinum phase was estimated to be 1,700° C, which was the approximate melting point of a 74.6 a/o platinum alloy as determined from optical pyrometer measurements.

In the composition range of 75 to 84 a/o platinum, the eutectic horizontal at 1,345° C was not observed, although a peritectic reaction was detected near 1,460° C. An alloy of 84.5 a/o platinum developed thermal arrests at 1,340°, at 1,460°, and at its melting temperature of 1,481° C, thus indicating that the alloy must lie in the eutectic field adjoining the terminal solid solution field but still be below the peritectic horizontal. An alloy of 82.8 a/o platinum did not develop the eutectic arrest, but it did show a definite arrest at 1,458° C, thus indicating that a phase boundary must lie between the 82.8 and 84.5 a/o platinum compositions.

In the composition range of 75 to 100 a/o platinum, the minimum located near 1,345° C was determined to be due to a eutectic reaction, inasmuch as a thermal arrest was observed in alloys having nominal compositions in the range 84.5 to 94.6 a/o platinum. No other arrests were observed below this temperature in this composition range.

The eutectic horizontal at the platinum-rich end was located by quenching techniques, using an alloy of 88.6 a/o platinum—the approximate eutectic composition. While this alloy showed no fusion at 1,340° C, it both fused and shattered on quenching from 1,350° C. This behavior established the fusion temperature within the range observable in this procedure, and thereby confirmed the results of thermal analysis of alloys of 84 to 95 percent.

### 5.2. Microstructures of the Alloys

**Figure 1:**
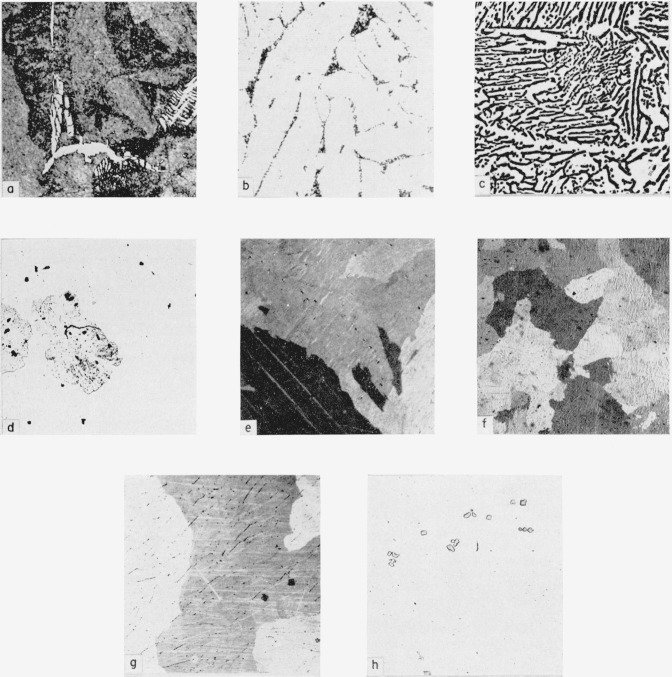
Microstructures of uranium-platinum alloys Samples a, b were etched in chromic acid; c, d, e, f, g were electropolished in solution of orthophosphoric acid, ethylene glycol, and ethyl alcohol; h was not etched.
Alloy of 5.0 a/o platinum, quenched from 720° C, UPt in uranium matrix. × 500.Alloy of 2.35 a/o platinum, after thermal analysis, UPt in uranium matrix. × 1000.Alloy of 2.35 a/o platinum, quenched from 700° C, UPt in uranium matrix. × 200.Alloy of 1.2 a/o platinum, quenched from 590° C, UPt in uranium matrix. × 100.Alloy of 1.2 a/o platinum, quenched from 600° C, uranium solid solution. × 500.Alloy of 0.9 a/o platinum, homogenized, uranium solid solution. × 100.Alloy of 1.2 a/o platinum, after thermal analysis, UPt in uranium matrix. Polarized light. × 100.Alloy of 49.8 a/o platinum, uranium in UPt matrix. × 200.

The microstructures of alloys containing less than 5 a/o platinum varied appreciably depending on quenching temperature, and thus served to indicate the solubility variations in the several allotropic phases of uranium. The microstructure of the 5.0 a/o platinum alloy aided in determining the approximate maximum solid solubility of platinum in gamma-uranium, for when quenched from 720° C, the structure of this 5.0 a/o platinum alloy was two phase (fig. 1a). The second phase was observed to be spherodized at 990° C and had almost entirely disappeared, thereby indicating that this composition closely approximated the limit of solid-solubility of platinum in gamma-uranium. Thus it was concluded that the limit of solubility at the eutectic temperature was approximately 5 a/o platinum. The alloy of 2.35 a/o platinum varied in appearance according to the quenching temperature. For example, the structure of a slowly-cooled sample (3° C/min) was two-phase (fig. 1b); whereas quenching from 590° C produced what appeared to be a transition structure, and quenching from 700° C produced a structure which appeared to be almost entirely of the eutectoid type (fig. 1c). Furthermore, the structures of samples of this alloy quenched from temperatures of 720° C and above were of the solid solution type. It therefore appears that this composition (2.35 a/o platinum) closely approaches the eutectoid of the gamma decomposition.

The beta-uranium solvus was located approximately by quenching experiments. A 1.2 a/o platinum alloy, when quenched from 590° C, revealed what appeared to be a eutectoid decomposition (fig. 1d) but became a single phase structure when quenched from 600° C (fig. 1e). On the other hand, the 2.35 a/o platinum alloy was two-phase both at 590° C and at 700° C, thus locating the beta solvus between these two compositions. The room temperature solubility of platinum in uranium was shown to lie between 0.9 and 1.2 a/o platinum (figs. 1f, g). Even though thermal analysis indicates that the platinum solubility in alpha-uranium may be below 0.5 a/o, consideration of homogenized and alpha-quenched specimens indicates the higher solubility of between 0.9 and 1.2 a/o platinum.

The solidus line in the uranium-rich field was located by quenching techniques. An alloy of 1.2 a/o platinum showed evidence of melting in the temperature range between 1,060° C and 1,090° C. For an alloy of 2.35 a/o platinum the solvus lies between 1,050° C where solid solution was evident and 1,075° C, where fusion occurred.

Metallographic evidence for the eutectic temperature was sought by using a 13.1 a/o platinum alloy which was estimated to be near the eutectic composition. A sample of this composition, on being quenched from 1,000° C, showed a eutectic structure while another such sample, quenched from 1,010 °C, showed partial fusion, thus bracketing the eutectic horizontal within ±5° C at 1,005° C.

The homogenized specimens in the composition range of 0 to 50 a/o platinum showed an increasing amount of second phase with increasing platinum content. The two phases present in the homogenized 33.6 a/o platinum alloy and their relative amounts indicated that no compound of 33.3 a/o platinum exists. Both of the phases were optically active with the terminal solid solution showing fourfold activity and the second phase showing twofold activity under polarized light. The microstructure of the 49.8 a/o platinum specimen was practically single phase (fig. 1h). In view of the structures observed in the two ranges of 0 to 49.8 a/o platinum and of 49.8 to 60.4 a/o platinum, it was concluded that a phase existed at the apparent composition of UPt.

**Figure 2:**
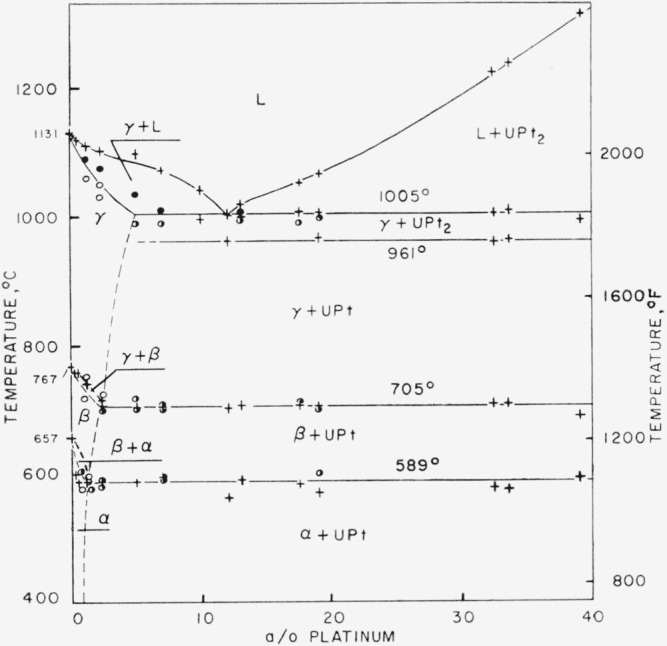
Uranium-rich section of uranium-platinum phase diagram ○ indicates single phase alloy, ◑ indicates two phase alloy, ● indicates fusion; + indicates thermal arrest.

The metallographic and thermal data for the uranium-rich alloys have been summarized in figure 2.

Instead of the expected two-phase structure, alloys in the range of 55 to 64 a/o platinum showed a single phase structure after homogenization. However, quenched specimens in this range showed increasing amounts of silica contamination upon quenching from higher temperatures. Since alloys in this range had also showed a reaction with beryllia when induction melted, these alloys were protected from contact with silica by molybdenum sheet. After homogenization these alloys then showed two phases, consistent with their “as cast” structure. In addition, the X-ray patterns were also consistent with the observed microstructures and indicated no apparent molybdenum contamination.

**Figure 3:**
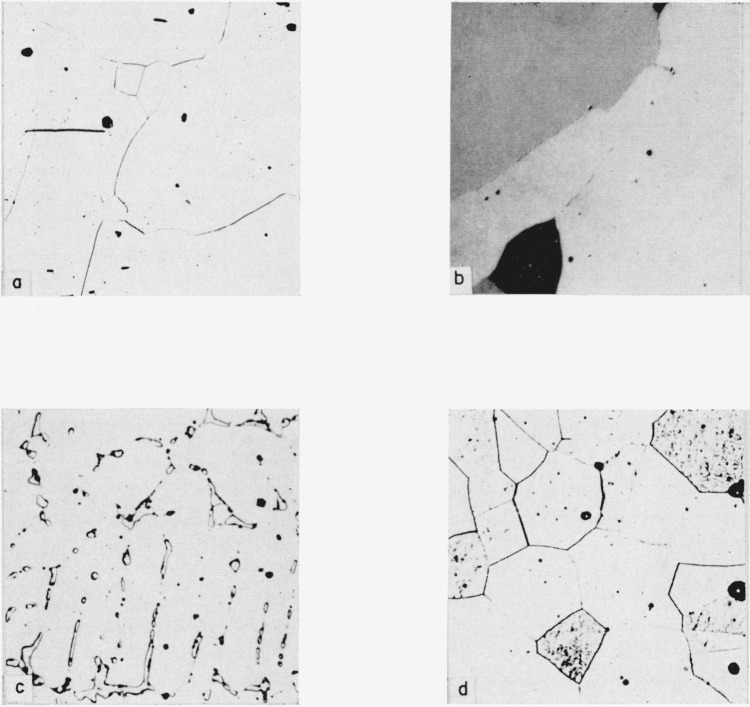
Microstructures of uranium-platinum alloys Specimens a, d were etched in sodium cyanide; b, c were not etched.
Alloy of 66.7 a/o platinum, UPt_2_ phase. × 100.Alloy of 74.6 a/o platinum, UPt_3_ phase. Polarized light, × 500.Alloy of 82.8 a/o platinum, UPt_3_ in a matrix of UPt_5_. × 500.Alloy of 96.4 a/o platinum, platinum terminal solid solution. × 250.

The two phase structures occurring in the alloys between 50 and 75 a/o platinum showed one optically inactive phase, one that revealed twofold optical activity, and one that revealed fourfold optical activity under polarized light. In the alloys between 50 and 64 a/o platinum the phase of twofold activity (UPt) and an optically inactive phase were present, and the alloys in the range of 68.2 to 74.6 a/o platinum contained an optically inactive phase and one of fourfold activity. The presence of the phase having no optical activity was interpreted to signify that a new homogeneous “field” was to be encountered between 63.7 and 68.2 a/o platinum. The distribution of the constituents of the microstructures indicated the phase boundary to have the apparent composition of UPt_2_. In confirmation of this, a single phase structure was developed in an alloy of 66.7 a/o platinum (fig. 3a) when heated by its own electrical resistance to about 1,350° C, thus serving to substantiate the existence of the UPt_2_ compound.

In the composition range of 75 to 96 a/o platinum three phases were identified. However, alloys in this range reacted readily with silica tubing and consequently had to be protected with beryllia during quenching studies. Alloys in this range also exhibited varying degrees of optical activity under polarized light. The structure of the alloy at 74.6 a/o platinum consisted almost entirely of a single phase having fourfold optical activity (fig. 3b), thus apparently indicating the existence of the UPt_3_ phase. Alloys in the composition range 84 to 95 a/o platinum were two phase, consisting of the terminal solid solution which was not active under polarized light and a second phase which displayed twofold activity under polarized light. The 84.5 a/o platinum alloy showed an active and an inactive phase under polarized light, and it consisted predominantly of the active phase. The 80.0 a/o platinum alloy consisted of two phases both of which were active under polarized light, one being twofold and the other fourfold active. In view of this, it was concluded that an intermetallic compound must be located between 80.0 and 84.5 a/o platinum; of the two possible compound compositions in this range, UPt_4_ and UPt_5_, that of UPt_4_ must be eliminated since the 80.0 a/o platinum alloy definitely consisted of two phases. Also, since an alloy of 82.8 a/o platinum was predominantly single phase (fig. 3c) and showed twofold activity under polarized light, it would seem to indicate proximity to the nominal phase composition. Thus the existence of the UPt_5_ (83.3 a/o Pt) phase was postulated, and its existence persisted to at least 1,340° C, as indicated from observation of quenched specimens. In the range of 75 to 83.3 a/o platinum (UPt_3_ to UPt_5_) only two phases were observed, both being optically active.

The solid solubility of uranium in platinum was determined by metallographic observation of alloys containing more than 94 a/o platinum. An alloy of 96.4 a/o platinum was entirely single phase (fig. 3d) whereas a 94.6 a/o platinum alloy showed a small amount of second phase, from which it may be concluded that the room temperature solubility of uranium in platinum is approximately 4 a/o. At elevated temperatures the 96.4 a/o platinum alloy was single phase up to the maximum quenching temperature of 1,340° C, and the 94.6 a/o platinum alloy contained two phases to the same temperature. Thus, these quenching experiments indicate that the solvus lies between 94.6 and 96.4 a/o platinum up to 1,340° C.

### 5.3. Microhardness Results

Microhardness tests were made to assist in the differentiation between the various constituents in the several fields occurring in the uranium-platinum system (table 3).

Hardness data provided additional evidence which helped to clarify the peculiarities noted in an alloy of 54.8 a/o platinum. After homogenization in silica tubing, samples of this alloy showed a single phase having a diamond pyramid number of 565. However, after homogenization using a protective molybdenum sheet, the samples exhibited two phases having hardnesses of DPH 419 and 822 which were comparable to the hardnesses of the two phases present in this alloy after a thermal analysis run. The hardnesses of phases in alloys containing from 33.6 to 74.6 a/o platinum served to substantiate the conclusion that the alloy-refractory reaction resulted in the peculiarities noted in the 54.8 a/o platinum alloy.

### 5.4. X-Ray Data

X-ray diffraction patterns were obtained from the same specimen surfaces that were used for the metallographic examinations, and the data obtained were correlated with the microstructures. The characteristic *d*-values of the intermetallic phases are listed in table 4. The alloys in the composition range 75 to 85 a/o platinum were very brittle and could be readily crushed to powder; hence, camera techniques were employed to supplement the diffractometer data.

X-ray patterns were analyzed and compared with the known diffraction patterns for uranium [6, 7, 8], with the pattern for platinum [9], and with the reported UPt_3_ pattern [3].

The diffraction patterns for the homogenized specimens in the range of 0 to 49.8 a/o platinum showed, with increasing platinum content, decreasing relative intensity of lines and in the number of lines attributable to the terminal uranium phase, as well as in the appearance of additional lines which could not be ascribed to any previously reported compound. Alloys containing more than 49.8 a/o platinum had no lines corresponding to the uranium phase but did have lines believed to be caused by presence of the compound UPt which existed at 50 a/o platinum. The lines obtained from the UPt phase had almost disappeared in an alloy containing 63.7 a/o platinum, and a still different pattern attributable to another phase had become apparent. In an alloy of 68.2 a/o platinum the UPt lines had disappeared, being replaced by the lines of a new phase. These results indicated the existence of a phase containing 66.7 a/o platinum, UPt_2_. An alloy of 74.6 a/o platinum had lines believed to be obtained from UPt_3_ and none that could be assigned to either the UPt_2_ or the platinum-rich terminal solid solution. As the amount of platinum increased beyond 75 a/o platinum, the UPt_3_ pattern became weaker, and a new pattern, that attributed to the UPt_5_ phase, appeared. The presence of the UPt_5_ phase was indicated in the patterns from alloys of up to 82.0 a/o platinum. Progressive changes in the powder patterns of alloys having 76.0, 83.0, and 84.5 a/o platinum clearly indicated the presence of a compound having the apparent UPt_5_ composition. In the range between 84.5 and 96.4 a/o platinum the characteristic *d*-values of the terminal solid solution were observed in conjunction with a set of *d*-values which had also been associated with the UPt_5_ values determined by camera techniques.

Diffraction patterns were also obtained from alloys having essentially a single phase. In most cases the patterns exhibited strong, sharp diffraction lines, although the pattern from the 49.8 a/o platinum alloy (UPt phase) was very weak and poorly defined. No special effort was made to determine crystal structures. However, it was apparent that the characteristic *d*-values of the UPt_3_ phase obtained from these experiments did not coincide with the calculated values for the proposed UPt_3_ compound [3]. The discrepancies may be attributed to the different methods of preparing the samples.

The solid solubility of uranium in platinum was determined from quenched specimens by means of back-reflection X-ray methods. The film-to-specimen distance was calibrated by using the known “reflections” from the 420 plane of silver in the powder form. The change of lattice parameter with the increase of uranium solubility is positive (table 5). From these data, the solubility of uranium in platinum increases from a value of 4.0 a/o at room temperature to a maximum of 4.5 a/o at the eutectic temperature. The calculated increase in volume of the platinum lattice at the eutectic temperature is a maximum of 3 percent.

The X-ray data obtained from quenched specimens containing low platinum percentages, 0.25 to 5.0 a/o platinum, were compared with the data for the uranium phases. There was no evidence of retention of the beta-uranium or gamma-uranium phases in the diffractometer patterns.

## 6. Discussion

### 6.1. Uranium Terminal Solid Solution

The degree of solubility of platinum in uranium was determined primarily by metallographic data. The microstructures of alloys containing greater than 5 a/o platinum consisted of two phases when quenched from near the eutectic temperature. The 5.0 a/o platinum alloy quenched from 990° C contained only a trace of a second phase. Hence, it was concluded that the maximum solubility of platinum in uranium at the eutectic temperature was approximately 5 a/o. The structure of a quenched beta alloy having 2.35 a/o platinum was typically eutectoid and thus located the gamma-uranium decomposition, and the structure of this alloy quenched from the gamma field was typically solid solution. The 0.5 a/o platinum alloy was solid solution throughout the beta field. However, a 1.2 a/o platinum alloy was two phase at 590° C and solid solution at 600° C. The 2.35 a/o platinum alloy was two phase both at 590° C and at 700° C. Hence, the maximum solubility of platinum in beta-uranium lies between 1.2 a/o and 2.35 a/o. The room temperature solubility of platinum in uranium was shown to lie between 0.9 and 1.2 a/o platinum.

X-ray data of alloys containing up to 5 a/o platinum, obtained from quenched specimens, gave no conclusive evidence of the retention of beta- or gamma-uranium, as previously reported for this system [3].

### 6.2. Uranium-UPt_2_ Eutectic

Thermal analysis data indicated a decrease in the melting point of uranium alloys to a minimum at approximately 1,005° C at about 12 a/o platinum. The existence of a reaction horizontal was noted by thermal analysis of alloys in the composition range of 10 to 39 a/o platinum. A metallographic survey of quenched alloys in the composition range of 5 to 50 a/o platinum indicated the existence of a eutectic horizontal at approximately 1,005° C. The application of lever analysis to the distribution of the microconstituents indicated the placement of the eutectic point at about 12 a/o platinum. Based on these data, it was concluded that a eutectic between the terminal solid solution and UPt_2_ exists at an apparent composition of 12 a/o platinum at a temperature of 1,005° C.

### 6.3. UPt Phase

Thermal analysis data show the presence of the arrests characteristic of the allotropic transformations of uranium in the composition range of 0 to 45 a/o platinum, and the data fail to show the presence of these arrests in alloys in excess of 50 a/o platinum. Also, when this information is coupled with the presence of an arrest at 961° C, it must be considered indicative of a compound formed by a peritectoid reaction at 961° C and 50 a/o platinum. Since the 49.8 a/o platinum alloy was essentially single phase after homogenization while the 33.6 a/o and 39.0 a/o platinum alloys were definitely two phase, the most likely composition for a compound was in the 1 to 1 atomic ratio. X-ray techniques utilizing the disappearing phase method also indicate the presence of a phase boundary at 50 a/o platinum. Observations of the specimens under polarized light indicated two active phases in the 0 to 50 a/o platinum range and only one active phase in the field between 50 and 66 a/o platinum. Based on all these data, it was concluded that the compound formed peritectoidally at 961° C has the apparent composition of UPt.

### 6.4. UPt_2_ Phase

X-ray data for alloys in the range 55 to 75 a/o platinum indicated the presence of a phase in the vicinity of 67 a/o platinum. The increasing strengths of certain diffraction lines and the comparison of the *d*-values for the UPt and UPt_3_ phases permitted the separation of the pattern for the UPt_2_ phase.

The microstructures of the alloys in the composition range 66 to 75 a/o platinum contained two phases, one of which showed fourfold activity under polarized light and a second which was not active. This differed from two phase alloys in the 50 to 66 a/o platinum composition range, for these showed one constituent very strongly twofold active and a second constituent not optically active. Thus the metallographic data indicated a change of the phases being located near 67 a/o platinum. The single phase microstructure developed in a 66.7 a/o platinum alloy heated by its own electrical resistance completely substantiated the conclusion that the UPt_2_ phase was present.

In view of these data, it was concluded than an intermetallic compound UPt_2_ exists and thermal analysis data indicate that it is formed peritectically at 1,370° C.

### 6.5. UPt_3_ Phase

The alloy having 74.6 a/o platinum had a melting point of 1,700° C, the highest observed in the melting point determinations. This was indicative of a congruently melting compound situated near the 75 a/o platinum composition. The X-ray data revealed that a phase boundary should exist at 75 a/o platinum, for the data showed that one specific pattern became easily identifiable as the 75 a/o platinum content was approached. The essentially single phase microstructure of a homogenized 74.6 a/o platinum alloy indicated a possible phase boundary. In addition, a change of optical activity under polarized light was apparent near the 75 a/o platinum composition. In view of these data, it was concluded that UPt_3_, a congruently melting compound, exists in this system.

### 6.6. UPt_5_ Phase

Alloys in the neighborhood of the stoichiometric UPt_5_ composition were extremely reactive, being contaminated by either silica or molybdenum. These reactions completely masked UPt_5_ on the preliminary survey though certain inconsistent data were noted. Protection of the alloys with beryllia permitted heat treatment without contamination. The compound was eventually located by thermal analysis data in that the 82.8 a/o platinum alloy did not develop a thermal arrest at 1,340° C while the 84.5 a/o platinum alloy did show such an arrest. However, both of these alloys did develop an arrest near 1,460° C. Metallographic data in the UPt_5_-terminal solid solution field showed that the UPt_5_ had twofold activity under polarized light while the terminal solid solution revealed no activity. In the two phase field of alloys of 75 to 83 a/o platinum one of these phases showed twofold activity, whereas the other displayed fourfold activity under polarized light. The microstructure of the alloy at 82.8 a/o platinum was predominantly single phase and showed twofold activity under polarized light. Thus the metallographic data indicated the existence of a compound at approximately 83 a/o platinum. Both X-ray diffractometer data obtained from the metallographic specimens and the diffraction data obtained from unsieved powder specimens indicated, by the disappearing phase method, the existence of a compound at UPt_5_. This, coupled with the existence of a reaction horizontal at 1,460° C, indicated the existence of UPt_5_ which forms peritectically at 1,460° C.

### 6.7. Platinum Terminal Solid Solution

Results of both the X-ray and metallographic examinations show that the solvus of the platinum terminal solid solution rises very steeply from room temperature to the eutectic temperature. The high temperatures involved and the economics of platinum alloy studies did not permit more extensive alloy preparation with the resultant more precise location of the solvus. However, the X-ray and metallographic data, coupled with the thermal analysis data, locate the solvus within the composition range of 94.6 to 96.4 a/o platinum for the temperature range from room temperature to 1,340° C. In view of this, the solubility of uranium in platinum is considered to be 4.0 a/o at room temperature, increasing to 4.5 a/o at 1340° C.

A combination of thermal and metallographic data fixed the eutectic composition near 87.5 a/o platinum and at 1,345° C.

## 7. Summary

**Figure 4:**
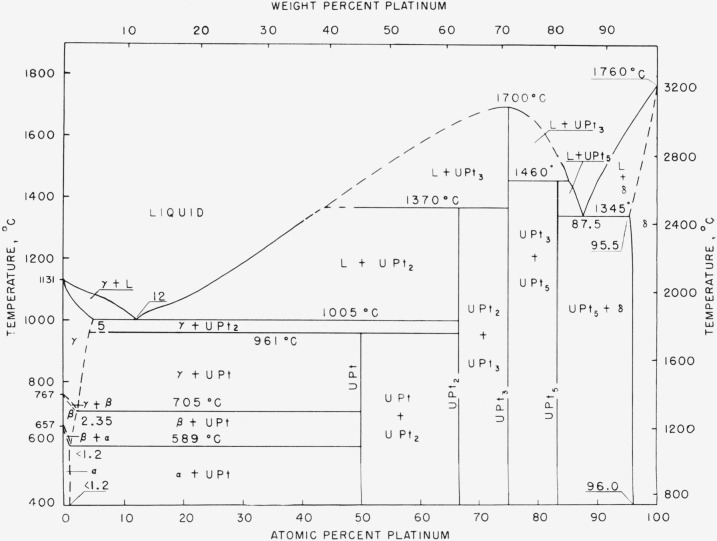
Uranium-platinum phase diagram.

The platinum-uranium system (fig. 4) was constructed from data obtained by thermal, metallographic, and X-ray analysis.

This system is characterized by two eutectics, one occurring at 1,005° C and a composition of 12 a/o platinum and the second at 1,345° C and 87.5 a/o platinum; and four intermetallic compounds, one formed peritectoidally at 961° C with an apparent composition of 50 a/o platinum (UPt), the second formed peritectically at 1,370° C with an apparent composition of 66.7 a/o platinum (UPt_2_), the third melting congruently at about 1,700° C with an apparent composition of 75 a/o platinum (UPt_3_), and the fourth formed peritectically at 1,460° C with an apparent composition of 83.3 a/o platinum (UPt_5_).

The solubility of uranium in platinum is approximately 4.5 a/o at the eutectic temperature of 1,345° C, decreasing with temperature to about 4.0 a/o at room temperature. The solubility of platinum in uranium is approximately 5 a/o at the eutectic temperature of 1,005° C, decreasing to near 2.35 a/o at the gamma-beta transformation; it is less than 1.2 a/o at the beta-alpha transformation, and is greater than 0.9 a/o at room temperature.

The gamma-beta transformation temperature of uranium was lowered from 767° to 705° C, and the beta-alpha transformation temperature was lowered from 657° to 589° C by the addition of platinum.

## Figures and Tables

**Table 1:** Chemical composition of the uranium-platinum alloys

Alloy No.	Uranium	Platinum	

	*Wt %*	*Wt %*	*Atomic %*
P–1	99.8	0.2	0.25
P–2	99.6	.4	.5
P–25	99.3	.7	.9
P–16	99.0	1.0	1.2
P–17	98.1	1.9	2.35
P–26	95.9	4.1	5.0
P–15	94.2	5.8	7.0
P–40	91.7	8.3	10.0
P–39	89.8	10.2	12.1
P–13	89.0	11.0	13.1
P–18	85.0	15.0	17.7
P–4	83.7	16.3	19.2
P–20	71.9	28.1	32.3
P–19	69.7	29.3	33.6
P–37	65.8	34.2	39.0
P–29	60.1	39.9	44.7
P–27	55.1	44.9	49.8
P–12	50.1	49.9	54.8
P–3	46.4	53.6	58.8
P–30	44.8	55.2	60.4
P–11	41.0	59.0	63.7
P–38	38.0	62.0	66.7
P–28	36.2	63.8	68.2
P–10	29.4	70.6	74.6
P–31	28.4	71.6	76.0
P–32	23.6	76.4	80.0
P–21	23.1	76.9	80.3
P–9	21.2	78.8	82.0
P–33	20.3	79.7	82.8
P–22	20.2	79.8	83.0
P–34	18.5	81.5	84.5
P–23	16.2	83.8	86.3
P–14	13.6	86.4	88.6
P–8	9.1	90.9	92.4
P–24	6.5	93.5	94.6
P–7	4.4	95.6	96.4

**Table 2:** Summary of thermal data

Composition, *a*/*o Pt*	Thermal arrests					

	a. Pt versus Pt—10% Rh thermocouple					
	
	Liquidus	UPt_5_	Eutectic	UPt	*γ*→*β*	*β*→*α*
	
	*°C*	*°C*	*°C*	*°C*	*°C*	*°C*
0.0	1131	……….	……….	……….	767	657
.25	1131	……….	……….	……….	759	599
.5	1120	……….	……….	……….	759	589
1.2	1110	……….	……….	……….	741	588
2.35	1102	……….	……….	……….	715	589
5.0	1098	……….	……….	……….	703	597
7.0	1072	……….	……….	……….	705	596
10.0	1043	……….	996	……….	697	579
12.1	1005	……….	1005	961	702	563
13.1	1020	……….	999	……….	706	590
17.7	1051	……….	1009	……….	705	583
19.2	1066	……….	1005	967	704	567
32.3	1222	……….	1002	959	708	576
33.6	1239	……….	1009	963	709	571
39.0	……….	……….	993	953	689	591
44.7	1385	……….	1030	963	734	……….
84.5	1481	1460	1340	……….	……….	……….
86.3	……….	……….	1336	……….	……….	……….
	
	b. Optical pyrometer					
	
39.0	a1310	……….	……….	……….	……….	……….
49.8	1415	……….	……….	……….	……….	……….
54.8	1515	……….	……….	……….	……….	……….
60.4	1385	……….	……….	……….	……….	……….
68.2	1360	……….	……….	……….	……….	……….
74.6	1700	……….	……….	……….	……….	……….
	
	c. W–Mo thermocouple					
	
80.3	1655	……….	……….	……….	……….	……….
82.0	1548	1455	……….	……….	……….	……….
82.8	1525	1458	……….	……….	……….	……….
88.6	1545	……….	1360	……….	……….	……….
94.6	1550	……….	1363	……….	……….	……….
96.4	1660	……….	……….	……….	……….	……….
99.5	1760	……….	……….	……….	……….	……….

a Liquidus or reaction temperature.

**Table 3:** Hardness of phases in uranium-platinum alloys

	Diamond pyramid hardness^a^

Uranium solid solution	425
UPt	385
UPt_2_	905
UPt_3_	405
UPt_5_	610
Platinum solid solution	250

a Average of 5 or ore determinations.

**Table 4:** X-ray diffraction data of uranium-platinum intermetallic compounds

UPt		UPt_2_		UPt_3_		UPt_5_	

*d*	*I*^a^	*d*	*I*	*d*	*I*	*d*	*I*

*A*		*A*		*A*		*A*	
2.80	*w*	2.93	*w*	3.14	*w*	2.60	*m*
2.76	*m*	2.33	*w*	2.72	*vw*	2.23	*s*
2.65	*w*	2.31	*w*	2.48	*m*	2.13	*m*
2.56	*vw*	2.26	*s*	2.44	*s*	2.06	*vw*
2.36	*s*	2.09	*m*	2.21	*s*	1.85	*w*
2.28	*w*	2.03	*w*	1.98	*vw*	1.70	*vw*
2.18	*w*	1.95	*w*	1.93	*w*	1.58	*vw*
2.11	*m*	1.84	*m*	1.74	*m*	1.513	*m*
2.00	*w*	1.77	*w*	1.69	*w*	1.426	*m*
1.92	*m*	1.65	*s*	1.435	*m*	1.173	*m*
1.85	*vw*	1.62	*vw*	1.364	*s*	1.131	*w*
1.78	*w*	1.49	*w*	1.308	*vw*	1.070	*vw*
1.72	*m*	1.44	*w*	1.253	*vw*	1.039	*vw*
1.66	*m*	1.280	*m*	1.230	*m*	1.005	*w*
1.64	*m*	1.247	*w*	1.223	*w*	……….	……….
1.56	*w*	1.032	*w*	1.206	*w*	……….	……….
1.52	*vw*	1.013	*w*	1.115	*vw*	……….	……….
1.471	*w*	0.985	*vw*	1.110	*m*	……….	……….
1.415	*s*	.970	*vw*	1.052	*w*	……….	……….
1.385	*m*	.906	*w*	0.990	*w*	……….	……….
1.347	*w*	……….	……….	.942	*w*	……….	……….
1.314	*w*	……….	……….	.933	*w*	……….	……….
1.277	*vw*	……….	……….	.925	*w*	……….	……….
1.270	*vw*	……….	……….	.912	*w*	……….	……….
1.192	*w*	……….	……….	……….	……….	……….	……….
1.181	*m*	……….	……….	……….	……….	……….	……….
1.166	*w*	……….	……….	……….	……….	……….	……….
1.147	*w*	……….	……….	……….	……….	……….	……….
1.103	*vw*	……….	……….	……….	……….	……….	……….
1.093	*w*	……….	……….	……….	……….	……….	……….
1.083	*vw*	……….	……….	……….	……….	……….	……….
1.070	*vw*	……….	……….	……….	……….	……….	……….
1.043	*vw*	……….	……….	……….	……….	……….	……….
1.006	*vw*	……….	……….	……….	……….	……….	……….
0.998	*w*	……….	……….	……….	……….	……….	……….
.978	*w*	……….	……….	……….	……….	……….	……….
.965	*w*	……….	……….	……….	……….	……….	……….
.954	*w*	……….	……….	……….	……….	……….	……….
.918	*w*	……….	……….	……….	……….	……….	……….

a *I* is intensity; *s—*strong; *m—*medium; *w—*weak; *vw*—very weak.

**Table 5:** Solubility determination of uranium in platinum

Quenching temperature	Lattice parameters, *A*				Resultant uranium solubility
	99.5 a/o Pt	96.4 a/o Pt	94.6 a/o Pt	92.4 a/o Pt	

° *C*					*a*/*o*
Room	3.925	3.953	3.956	3.956	4.0
1200	3.927	3.958	3.964	3.965	4.3
1340	3.927	3.958	3.966	3.967	4.5

## Footnotes

1 Figures in brackets indicate the literature references at the end of this paper.

2 Spectrochemical analysis of platinum: 0.01 to 0.1 percent of Pd and Be; 0.001 to 0.01 percent of Au; 0.0001 to 0.001 percent of Ca, Mg, and Si; and <0.0001 percent of Cu (all percentages by weight).
